# Quasi-Operando Liquid-Phase Electron Imaging of Metallic Copper Nanocubes Reveals Step-by-Step Subtle Dissolution, Redeposition, Reattachment, and Fragmentation Mechanisms during CO_2_ Electroreduction

**DOI:** 10.1021/acs.nanolett.6c00223

**Published:** 2026-04-07

**Authors:** Saltanat Toleukhanova, Petru Albertini, Vasiliki Tileli

**Affiliations:** † 27218 Institute of Materials, École Polytechnique Fédérale de Lausanne, Lausanne, CH−1015, Switzerland; ‡ Institute of Chemical Sciences & Engineering, École Polytechnique Fédérale de Lausanne, Lausanne, CH−1015, Switzerland

**Keywords:** metallic Cu nanocubes, CO_2_ reduction reaction, quasi-operando, TEM

## Abstract

Metallic copper nanocubes used in CO_2_ electroreduction (CO_2_ER) are known to exhibit moderate binding energies to intermediates and selectivity toward ethylene. However, the process by which they restructure, ultimately redistributing the active sites and causing deactivation, remains challenging to fully elucidate. Herein, we use electrochemical liquid-phase transmission electron microscopy to observe copper nanocube evolution during CO_2_ER in real time at nanometer resolution. Our statistical analysis reveals that dissolution/redeposition is the primary evolution mechanism. However, unlike other shapes, the thermodynamically active edges of copper cubes attract the redeposited aggregates that reattach to the cubes, moderating their surface-to-volume ratio. Additionally, the fragmentation mechanism was observed, which may occur due to highly defective sites. Our findings illustrate the synergistic effect of the thermodynamically high-energy sites and the role of the kinetic barrier in the dynamics of Cu nanocubes and their secondary aggregates, which affects their stability and as a consequence their selectivity over time.

## Graphical abstract

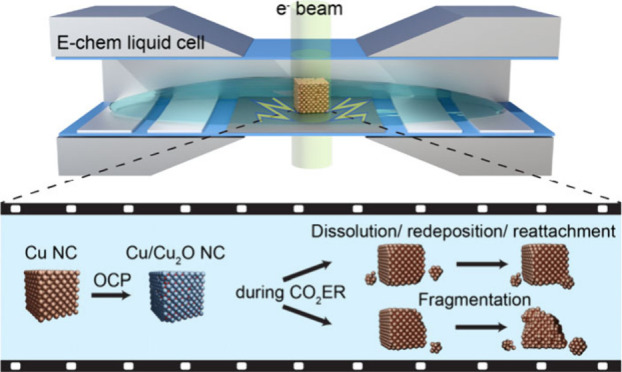

Copper is a uniquely effective catalyst material for CO_2_ electroreduction (CO_2_ER) with properties such as moderate binding energy toward reaction intermediates allowing for CO_2_ conversion into high-energy C_2+_ products.^1^ In addition, its facet-dependent selectivity toward hydrocarbon products^2^ such as CH_4_ and C_2_H_4_ along with the development of colloidal particle synthesis allowed preferentially oriented single crystal Cu catalysts to be used for targeted facet-dependent product selectivity.^3^ Moreover, the high surface area of faceted nanoparticles renders them more active than single-crystal bulk catalysts with the same exposed facets.^4^ Nevertheless, both faceted single-crystal nanoparticles and polycrystalline Cu catalysts eventually deactivate and lose selectivity toward CO_2_ER products during the reaction.^5^ Deactivation can occur for various reasons, including catalyst poisoning by impurities or reaction intermediates, or catalyst restructuring, which in turn redistributes active sites in the catalyst, rendering the catalyst inactive for CO_2_ER and instead increasing selectivity for the competing hydrogen evolution reaction (HER).^6–8^ Hence, understanding the deactivation mechanisms and conditions leading to it is of utmost importance for resolving the deactivation issue and designing highly selective catalysts with enhanced activity and long-term stability, paving the way for upscaling of the process.

There are intrinsic (i.e., nanocatalyst dimension, morphology, surface structure, and composition) and extrinsic (i.e., electrolyte pH, reaction cell type, and applied potential) factors that are altered during catalyst operation and which affect the overall degradation mechanisms.^9,10^ Among the different methods used nowadays to study various aspects of catalyst evolution over time,^11^ liquid-phase electron microscopy (LPEM) allows real-time monitoring of key properties such as structure, morphology, and size during restructuring.^12–14^ Most previous studies focused on Cu nanospheres and revealed spontaneous oxidation and further dissolution/redeposition mechanisms at the initial stages of CO_2_ER^15^ monitored in quasi-operando^12^ conditions, while other in situ studies showed nanograin formation, resulting in the generation of active sites that increased C_2+_ selectivity.^16^ In contrast, the degradation mechanism of ethylene (C_2_H_4_)-selective {100}-faceted Cu nanocubes (NCs) has been seldomly studied with LPTEM techniques. There exist in situ scanning TEM (STEM) studies of electrochemically synthesized Cu_2_O NC degradation at CO_2_ER-relevant conditions,^17,18^ which show the transition of single-crystal NCs into fragmented porous particles,^18^ while recent studies on Cu/Cu_2_O NCs indicated a three-step restructuring into polycrystalline metallic Cu nanograins.^19^ As was discussed earlier, along with other factors, the initial crystal structure and composition of the catalyst might alter the degradation mechanism. Hence, although Cu NCs are spontaneously oxidized on the surface upon immersion into electrolyte, the fully Cu_2_O NC or preoxidized Cu/Cu_2_O NC catalyst cannot be representative of metallic Cu NC degradation. Previously it was shown that metallic Cu NCs at different operation times undergo potential-induced nanoclustering followed by aggregation, with the transition time between these two mechanisms being dependent on the initial size of the Cu NCs.^8^ Another identical location TEM study also supports the adverse effect of high cathodic potentials on the restructuring of Cu nanocubes; however, nanoclustering was not observed.^20^

Herein, we focus on monitoring the evolution of single-particle, metallic Cu NCs at CO_2_ER-relevant potentials and directly follow step-by-step their deactivation/activation at startup and operation. To achieve this, we used Cu nanocubes that were synthesized with previously described colloidal chemistry protocols and that were reported to be highly selective for ethylene.^3,21^ Figure 1a demonstrates their overall well-faceted structure, while the projection area analysis of a representative sample of 129 NCs (shown in Figure S1) indicates that their size is approximately 38 ± 5 nm. The high-resolution high-angle annular dark-field (HAADF) STEM image and corresponding fast Fourier transform (FFT, insets of Figure 1b) confirm that the nanocubes are terminated by the (200) and (220) facets of the metallic Cu phase, which is also confirmed by chemical probing using electron energy loss spectroscopy (EELS, insets of Figure 1b).

**Figure 1:**
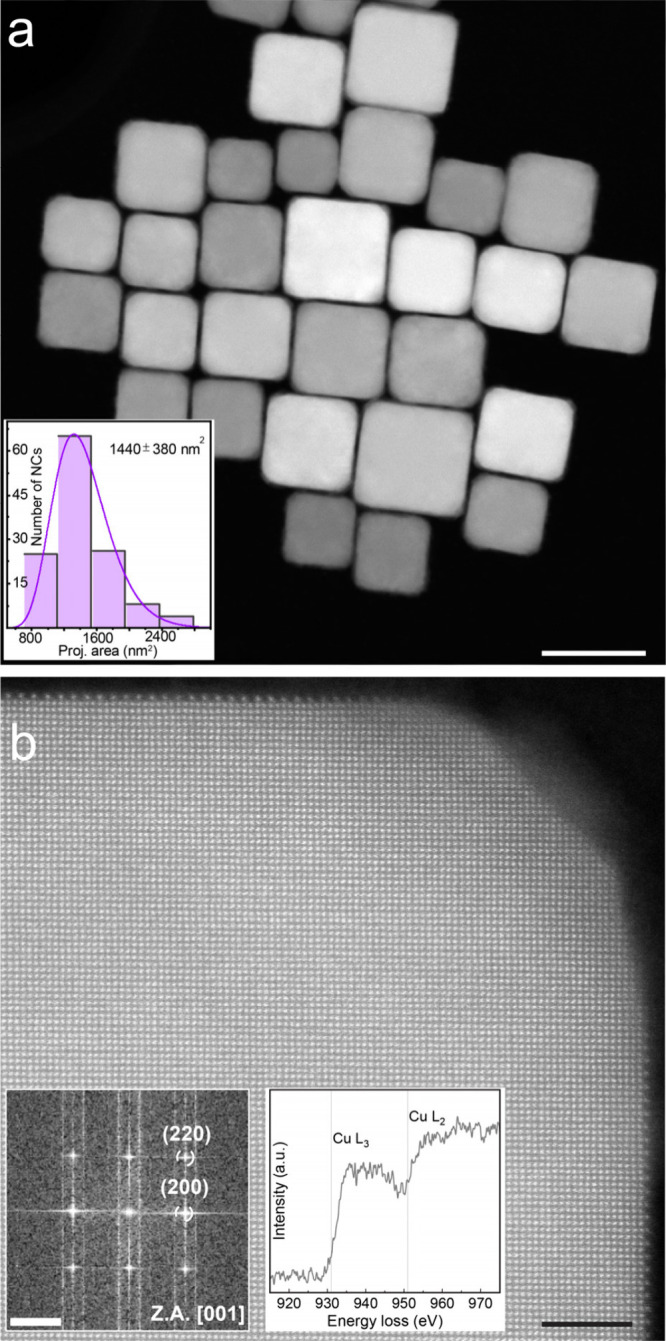
Characterization of primary Cu NCs. (a) HAADF STEM image of Cu NCs on a holey carbon grid. Scale bar, 50 nm. Inset: Histogram of the particle projection area distribution of Cu NCs. (b) High-resolution HAADF STEM image showing a quarter of a single Cu nanocube. Scale bar, 2.5 nm. Insets: Fast Fourier transform of (b), scale bar, 2 nm^–1^, and electron energy loss spectrum of the Cu nanocube showing the Cu L_3,2_ edge.

For the real-time imaging of the Cu cubes, we used energy-filtered electrochemical liquid-phase transmission electron microscopy (EF-LPTEM) while being attentive to keeping the electron dose to a minimum. Due to the small signal that can be attained under these conditions, advanced postprocessing was implemented to analyze the data. The complete pipeline is illustrated in Figure 2. In detail, the in situ EF-LPTEM setup, shown in Figure 2a, consisted of a cell assembled with a CO_2_ER-tailored electrochemical chip featuring a glassy carbon (GC) working electrode, which resulted in an expanded inert potential range of the substrate electrode. Dispersed Cu nanocubes were drop-cast onto the GC electrode overlaying the SiN_*x*_ membrane region (Experimental Methods). All measurements were performed in a CO_2_-saturated 0.1 M KHCO_3_ electrolyte solut. In situ imaging was done with an electron flux rate of 60 e^–^ nm^–2^ s^–1^ in energy-filtered TEM mode to enhance the contrast in the liquid cell,^22^ with a temporal resolution of 20 frames per second. Linear sweep voltammetry (LSV) to −0.8 V vs the reversible hydrogen electrode (RHE) followed by chronoamperometry (CA) was used to simulate the startup and working condition of the catalytic cell, respectively (Figure 2b). The uninterrupted recording was then synchronized with the electrochemical data. The raw image sequences exhibit low signal-to-noise, due to the use of a low electron dose to avoid beam-induced side reactions (Figure S2), and postprocessing was necessary. First, the data were denoised using the previously reported Cryo-CARE method based on noise-to-noise training.^23,24^ The advantage of this methodology is that it does not require ground truth data. Figure S3 presents the comparison between raw and denoised images along with the corresponding FFTs of each image. However, due to the workflow specifications, the resulting temporal resolution of the denoised data was 10 fps (Figure 2c). Within the scope of this catalyst system and the experimental conditions studied in this work, 10 fps was sufficient for seamless monitoring of the degradation processes. The full denoised image sequence along with the synchronized electrochemical data can be seen in Supplementary Movies S1–S5. The denoised image sequence was then segmented using the ImageJ plugin SAMJ^25^ to analyze the evolution of the particle projection area over time and potential (Figure 2d,e).

**Figure 2:**
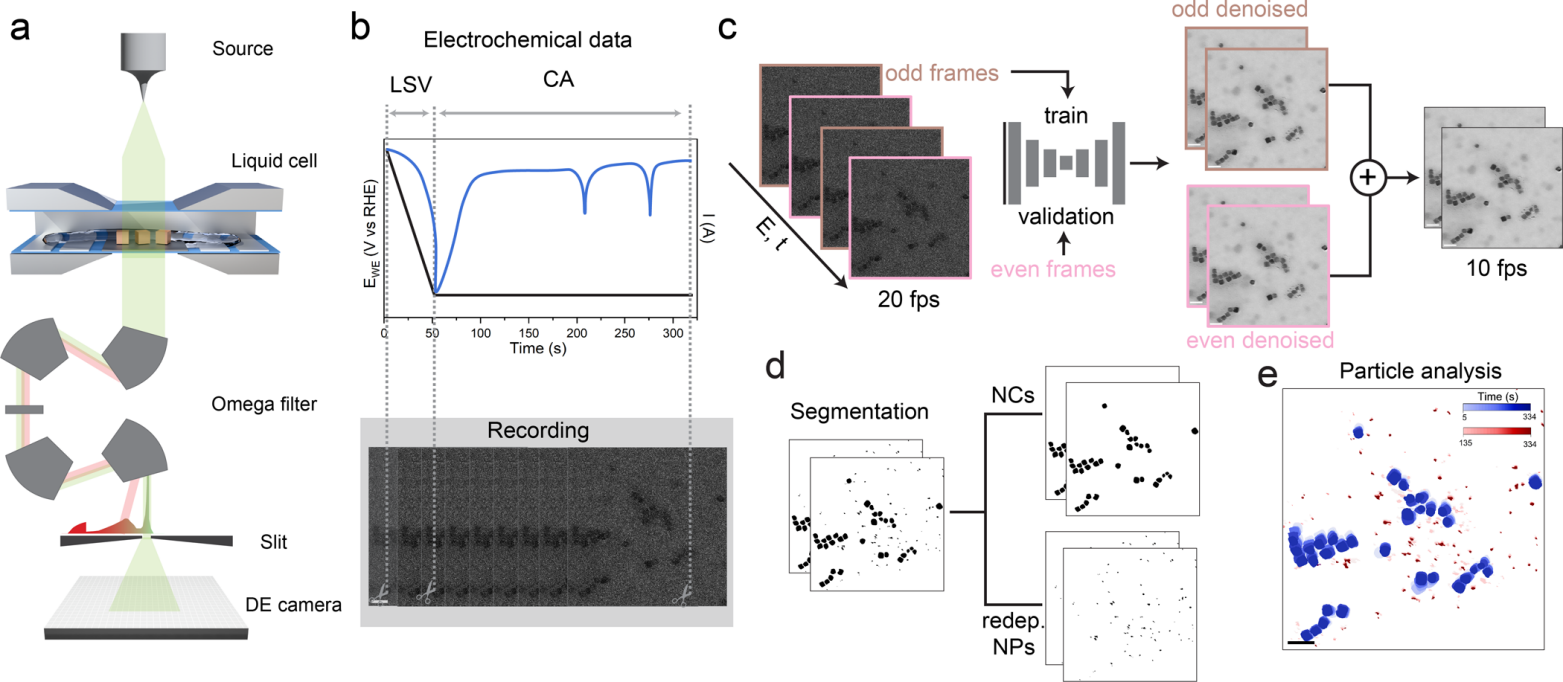
EF-LPTEM experimental workflow. (a) Main components of the microscope’s configuration, including the electrochemical liquid cell, Omega filter, and direct electron camera. (b) Illustrative electrochemical data and images demonstrating the synchronization of the electrochemical measurements and electron signal. (c) Denoising workflow including the training validation processes. (d) Segmentation of primary nanocubes and redeposited nanoparticles. (e) Color-coded segmented images of Cu NCs depicting their evolution over time during CO_2_ER.

Prior to the quasi-operando EF-LPTEM CO_2_ER measurements, we performed EFTEM imaging and selected area electron diffraction (SAED) of the NCs inside the liquid-containing cell. SAED showed diffused rings overlapping with the reference positions of the {200} plane of Cu_2_O and the {200} plane of the Cu phase (Figure 3a and Figure S4). Then, LSV and CA stimuli were applied (Figure 3b), followed by repeated imaging and SAED pattern acquisition after the measurement (Figure 3c and Figure S4c). When comparing the SAED patterns before and after, the absence of the diffused rings was evident after the experiment, indicating that the initial thermodynamically driven surface oxide (i.e., Cu_2_O) at the OCP was reduced at negative potential. Figure 3d depicts time-lapse images of the prevalent evolution mechanisms of the primary Cu cubes. The complete image sequence of the evolution of some representative nanocubes is shown in Supplementary Movie S2. The majority of the NCs undergo subtle dissolution followed by formation of aggregates that redeposit from the dissolved Cu ions. However, some appear only to dissolve during CA, others eventually exhibit fragmentation events, and others appear to regrow following attachment of secondary redeposited nanoparticles. The statistics of the four behavioral patterns are shown in Figure 3e, including the NCs that remain unaffected and are stable throughout the process, as identified with image postprocessing and analysis.

**Figure 3:**
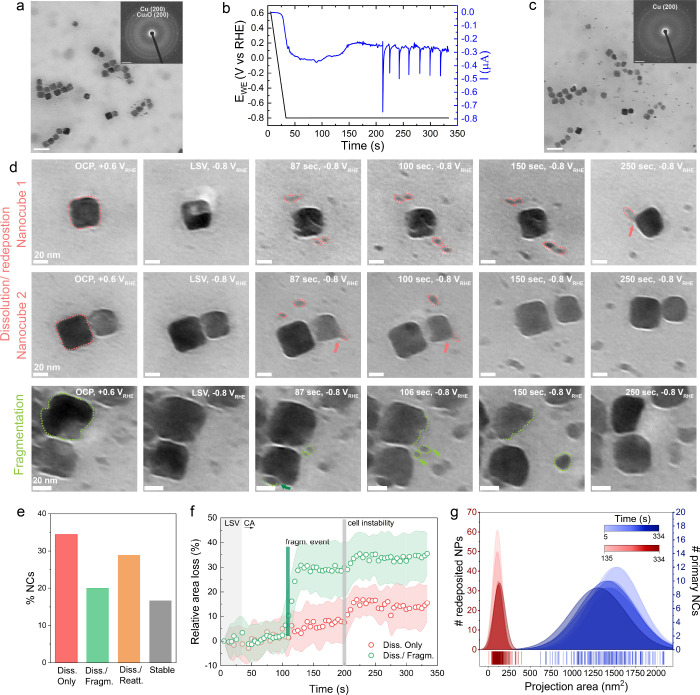
Evolution of Cu NCs at CO_2_ER. (a) In situ energy-filtered TEM image of Cu NCs at open circuit potential in 0.1 M KHCO_3_ electrolyte. Scale bar, 100 nm. Inset shows the corresponding selected area electron diffraction. Scale bar, 2 nm^–1^. (b) Current and potential profile versus time during linear sweep voltammetry and chronoamperometry measurements at −0.8 V vs RHE. (c) In situ energy-filtered TEM images of Cu NCs after electrochemical measurements. Scale bar, 100 nm. Inset depicts the corresponding electron diffraction. Scale bar, 2 nm^–1^. (d) Time-lapse images of Cu NCs synchronized with electrochemical data in panel (b), illustrating evolution of nanocubes during dissolution (upper two rows) and fragmentation degradation processes (bottom row) while redeposited nanoparticles (NPs) emerge. Pink arrows point to attachment events of redeposited NPs to Cu NCs. Dark green arrow indicates a fragment detaching from the Cu NCs. Green arrows point in the direction of detached fragments’ movement. (e) Bar plot illustrating the distribution of particles across different types of dissolution processes. (f) Relative projection area loss of Cu NCs as a function of time showing the trend of dissolution only and dissolution/fragmentation processes. Red and green shaded areas are standard deviations over ten and seven nanoparticles, respectively. (g) Distribution of projection area of primary Cu NCs and redeposited NPs plotted as a function of time. Panels (a), (c), and (d) show denoised images.

For the most part, our results indicate that during the startup regime of CO_2_ electroreduction, Cu nanocubes undergo mild dissolution (Figure 3d), which we attribute to a result from surface oxide reduction upon application of the cathodic potential sweep. During operation at a constant potential of −0.8 V vs RHE, the dissolved Cu ions in the electrolyte are reduced and grow into redeposited particles that are much smaller in size than the cubes and have rounded shape (Figure 3d). These particles tend to move around and coalesce with each other. In addition, some of the redeposited particles attach to the high-energy sites of the primary Cu nanocubes (i.e., to the edges, Figure S5). Measurements of local curvature for nanocubes undergoing attachment (Figure S6) show that redeposited particles tend to attach to regions of high curvature, though not necessarily at the point of maximum curvature on the particle. However, it should be noted that the lack of 3D information on the nanocubes may limit the precise identification of the preferential attachment sites, which could also be driven by mass transport limitations within the microcell. In post-mortem high-resolution HAADF STEM imaging of the sample after the in situ experiment in Figure 3 the crystal structure of the redeposited particles was found to be copper oxide, most likely due to air oxidation (Figure S7). Post-mortem characterization revealed fewer redeposits than were observed during the in situ measurements, possibly due to the dissolution of small, high surface energy redeposited particles back into the electrolyte when the cell is switched to OCP. Consistent with our findings, Liu et al. also reported the dissolution of Cu nanocubes with fewer observable redeposited particles after 20 min at −1.1 V vs RHE, as revealed by identical location TEM imaging.^20^ Furthermore, comparable restructuring behavior, characterized by the presence of redeposited and attached particles, was observed after 2 h of H-cell CO_2_ electroreduction at −1.1 V vs RHE (Figure S8). These bulk-cell experiments reveal that longer electrolysis times and higher cathodic potentials lead to further restructuring through sintering, while the measured Faradaic efficiencies were found to be consistent with previously reported values for Cu nanocubes of a similar size after the same duration of electrolysis.^8^ We note that previously reported extended-time bulk-cell experiments with 40 nm Cu NCs at the lower potential of −0.7 V vs RHE also show similar dissolved cubes, redeposits, and attached particles but without apparent sintering, suggesting that the high cathodic potential and long electrolysis times promote particle sintering. Meanwhile, we have also observed Cu nanocubes undergoing fragmentation (Figure 3d), happening via the detachment of fragments from the Cu nanocubes. These fragments then diffuse away from the cube and coalesce with the existing Cu nanoparticles.

Postanalytics of the sequence of images allows us to acquire information on the relative area loss of the dissolution and fragmentation mechanics, depicted in Figure 3e. The raw projection area measurements of 90 nanocubes are depicted in Figures S9–S12. Our results indicate that as a result of fragmentation the cubes lose around 30% of their projection area, as shown by the sharp change at around 115 s in Figure 3f. Our findings provide statistically robust experimental evidence for mechanisms that were earlier inferred only indirectly. Previously, Huang et al. reported a nanoclustering process similar to the degradation mechanism of Cu nanocubes during CO_2_ER.^8^ The authors suggested that nanoclustering is driven by the high cathodic potentials necessary for the CO_2_ER, as well as by the adsorption of CO_2_ and reaction intermediates. Interestingly, in our experiments we observe that nanocubes undergoing fragmentation have a more distorted shape (Figure S13); however, not all irregularly shaped nanocubes undergo fragmentation. Hence, we assert that fragmentation seems to occur randomly, presumably due to defects on the Cu facets, and statistically accounts for 20% of the behavioral pattern of the Cu cube evolution (Figure 3e). Additionally, fragmentation could be attributed to the formation of defects caused by structural changes between the oxidized and reduced version of the cubes, which could be unrelated to the catalytic reaction itself.

In contrast, the NCs that only exhibit dissolution, combined with the ones that dissolve and then appear to grow with attachment of redeposited NPs, are the prevailing mechanism of the evolution of Cu cubes, with more than 50% of the cubes exhibiting this behavior (Figure 3e). It is worth mentioning that our sample of Cu nanocubes had not only perfectly faceted nanocubes but also cubes with a higher fraction of undercoordinated sites and a small number of particles of irregular shapes. Interestingly, both a well-faceted cube and a cube with a significant number of undercoordinated sites underwent evolution through dissolution and redeposition, exhibiting a similar trend in their projection areas over the reaction time (Figure S14). Additionally, a comparison of the average dissolution rates of these two cubes shows that the defective cube, which has a higher fraction of undercoordinated sites, exhibits both higher dissolution and growth rates, indicating greater reactivity (Figure S15). Concerning the sharp increase at around 200 s, as seen in Figure 3f for the dissolved cubes, also indicated by the abrupt drop in the projection area (Figure S9, orange), we associate this with the spikes in the current profile (Figure 3b). These spikes are indicative of gas bubble formation, which disrupts the environment inside the microcell and eventually causes the movement of the surrounding liquid. This could result in off-axis rotation, tilting, or displacement of the Cu NCs, which would change the projection area of the cubes and could lead to misinterpretation of their evolution.^26^ We note that in the case of the NCs that undergo fragmentation, another relatively smaller jump in projection area loss (around 10%) was observed at the same time (200 s) as for Cu NCs undergoing dissolution. This confirms that this jump is caused by a disruptive event in the cell and is not associated with the catalytic reaction.

Finally, Figure 3g shows the projected area distribution of the cubes and redeposited nanoparticles over time. The broadening of both distributions is accompanied by a reduction in the mean area of the primary NCs and an increase in the mean area of the redeposited nanoparticles. Interestingly, the percentages of mean area loss and gain for the NCs and the redeposited nanoparticles are equal at 16%. Nevertheless, it is worth noting that, under electron imaging conditions, unaccounted mass loss is likely to occur for various reasons, including nanocube tilting and defocusing or undetected ion precipitation under different focusing conditions in the microcell, which would affect the segmentation result.

In addition to in situ TEM experiments, we performed square wave voltammetry (SWV) measurements on the bench before and after the LSV/CA protocol to electrochemically probe the restructuring of Cu nanocubes (Figures 4 and Figure S16). The experiments were conducted in CO_2_-saturated 0.1 M KHCO_3_ at −0.8 V vs RHE in an SEM liquid cell holder equipped with bulk counter (carbon) and reference (Ag/AgCl) electrodes to ensure the stability of the applied potential and to enable higher catalyst loading on a single GC electrode chip for improved signal detection. The SWV curve at the starting point displays three pairs of structure-sensitive and redox features (Figure 4 and Figure S16). The most intense peak pair at 0.03 and 0.13 V vs RHE corresponds to the adsorption of bicarbonate species onto the Cu {100} facets.^27,28^ The shoulder of the anodic peak between 0.13 and 0.2 V vs RHE indicates the adsorption of ionic species on defect sites, supporting the hypothesis on defect-induced fragmentation processes.^29,30^ The other two successive pairs of redox peaks, situated at more positive potentials, correspond to Cu^0^ ↔ Cu^+^ and Cu^+^ ↔ Cu^2+^.^31^ Interestingly, the SWV curve of the Cu nanocubes immediately after the LSV-CA measurement (Figure S16a) exhibits the same features but with lower peak current values and shifted bicarbonate adsorption/desorption features. The cathodic peak shifted slightly toward more negative potential values, while the anodic peak, which was at 0.13 V vs RHE, has a current that is around three times lower and has shifted positively to 0.2 V vs RHE. This results from the restructuring of the catalyst upon application of a constant cathodic potential.^27^ Additionally, the cathodic bicarbonate adsorption peak became broader, potentially related to surface order decay.^27^ Meanwhile, the Cu redox peaks retained their position on the potential axis and decreased only in the current value due to the preceding hold at the reducing potential.

**Figure 4:**
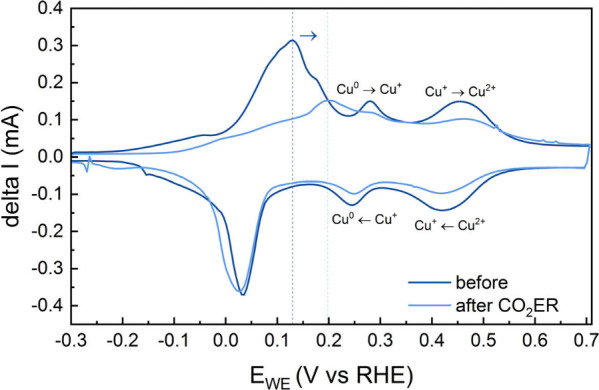
On-bench SWV measurements. Measured square wave voltammetry curves of Cu NCs before and after the CO_2_ER experiment.

Based on insights from the quasi-operando EF-LPTEM study, we summarize the life cycle of copper nanocubes at the early stages of the CO_2_ER in Scheme 1. The as-synthesized colloidal catalyst begins its life in the metallic state; however, due to the thermodynamic instability of the metallic Cu in aqueous media, the nanocubes are oxidized at OCP conditions in the electrolyte. Thereafter, during startup when the potential is swept from the OCP to reach the negative CO_2_ER operating potential (i.e., −0.8 V vs RHE), the surface oxide begins to reduce, resulting in the leaching of ionic Cu species into the electrolyte. At constant cathodic potential (during operation), these ionic species are reduced and nucleate when supersaturation is achieved and precipitate as redeposited nanoparticles. These redeposits are small in size and exhibit a round shape. Due to their high surface energy, aided by the low kinetic barrier due to the cathodic applied potential, they tend to coalesce with each other and some of the redeposited particles attach to the high-energy sites of Cu NCs. This mechanism, widely termed dissolution/redeposition, was previously reported for other Cu-based systems.^15^ However, the thermodynamic and kinetic profile of the faceted metallic nanocube surfaces distinguishes these systems from previous reports, as they ultimately result in the reattachment of the precipitated species to the primary cubes over time. In contrast, during fragmentation, Cu NCs undergo oxidation/reduction (i.e., dissolution/redeposition) only at the beginning of the reaction and then rapidly detach from the Cu nanoclusters. This process begins randomly, and it is expected to occur in nanocubes with cracks or defects, which become weak points during surface oxide reduction, triggering the detachment of Cu nanocube fragments. Finally, the detached fragments diffuse away into the electrolyte and coalesce with nearby redeposited particles.

**Scheme 1:**
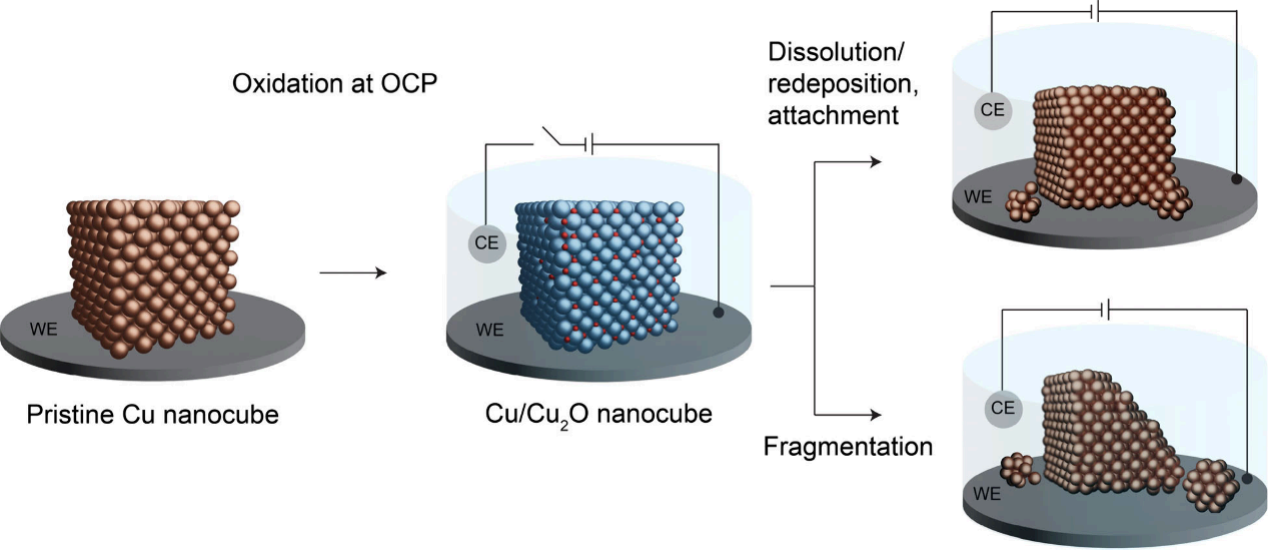
Summary of Evolution Mechanisms^a^

a Schematics summarizing the lifecycle of Cu NCs, from the as-synthesized state to open circuit potential in an assembled electrochemical cell, including the two major types of evolution mechanisms during CO_2_ER.

In summary, we reported on a methodology for performing energy-filtered liquid-phase TEM that does not disturb the electrocatalytic system, and on a pipeline for image processing that was used to reveal the step-by-step changes that Cu nanocube catalysts undergo under CO_2_ER. Our results unequivocally showed that during the initial stages of CO_2_ER, the majority of the nanocubes undergo subtle dissolution/redeposition, followed by the coalescence of the dissolved species to form secondary particles, some of which then attach to the high-energy sites of the primary cubes. Other cubes, presumably with defective sites, eventually fragment into statistically random events. By coupling real-time imaging with quantitative analysis, we move beyond qualitative observation to gain deeper mechanistic insight into the rectruring process. We envisage that further advances in characterization methodologies, such as real-time product detection during imaging, can aid toward the comprehensive understanding of Cu, and beyond Cu, electrocatalyst performance by linking the morphological and structural modifications, in other words their stability, directly to the evolution of their activity and selectivity over time.

## Supplementary Material













## Data Availability

The raw in situ data and denoising scripts are available at 10.5281/zenodo.18801651.

## Notes

Experimental methods, in situ EFTEM images, radial profile of SAED, projection area profiles for all analyzed NCs, time-lapse TEM image of different types of Cu NCs, LSV-CA of SWV of Cu NCs, captions of supplementary movies (PDF)
Movie S1: Denoised TEM image recording of Cu NCs during LSV-CA at −0.8 V vs RHE playback at 100 fps (MP4)
Movie S2: Denoised TEM image recording of Cu NCs during LSV-CA at −0.8 V vs RHE playback at 100 fps, scale bar 20nm (MP4)
Movie S3: Denoised TEM image recording of Cu NCs during LSV-CA at −0.8 V vs RHE playback at 100 fps, area 2 (MP4)
Movie S4: Denoised TEM image recording of Cu NCs during LSV-CA at −0.8 V vs RHE playback at 100 fps area 3 (MP4)
Movie S5: Denoised TEM image recording of Cu NCs during LSV-CA at −0.8 V vs RHE playback at 100 fps, area 4 (MP4)
