# Supplementary material for: Quasi-Operando Liquid-Phase Electron Imaging of Metallic Copper Nanocubes Reveals Step-by-Step Subtle Dissolution, Redeposition, Reattachment, and Fragmentation Mechanisms during CO2 Electroreduction
Source: Nano Lett. 2026 Apr 7;26(15):5094–101. doi: 10.1021/acs.nanolett.6c00223 (PMC13107515; doi:10.1021/acs.nanolett.6c00223)
Supplement: Supplementary file 1 [file nl6c00223_si_001.pdf]

Supplementary Information

Quasi-operando liquid-phase electron imaging of metallic copper nanocubes reveals step-by-step subtle dissolution, redeposition, reattachment and fragmentation mechanisms during CO_2 electroreduction

Saltanat Toleukhanova,[1] Petru Albertini,[2] & Vasiliki Tileli[1,*]

[1]Institute of Materials, École Polytechnique Fédérale de Lausanne, CH–1015 Lausanne, Switzerland

[2]Institute of Chemical Sciences & Engineering, École Polytechnique Fédérale de Lausanne, Switzerland

corresponding author : vasiliki.tileli@epfl.ch

Experimental methods

In situ liquid phase TEM

The in-house prepared electrochemical chip featured a glassy carbon (GC) working electrode, and Pt counter and reference electrodes. The quasi-reference electrode was calibrated versus Ag/AgCl bulk reference electrode, and the applied potential was converted to a reversible hydrogen electrode (RHE) scale using the Nernst equation. The bottom spacer chip was 100 nm (Hummingbird Scientific). Nevertheless, it is worth noting that membrane separation in conventional liquid cell is usually irrelevant to that of spacer thickness due to membrane bulging caused by the pressure difference across the membrane. Cu nanocubes' (40 nm in size) dispersion in Toluene (1500 ug/mL) was drop-casted with glass capillary onto the GC electrode overlaying the SiN_x electron-transparent membrane region. Due to the uncontrolled volume of the droplet within the capillary, the exact mass of the drop-casted catalyst cannot be calculated. Before the experiments, 0.1 M $KHCO_3$ electrolyte was purged with CO_2 gas for at least 1 hour, and before infusion of the electrolyte, its pH value was checked to be equal to that of the saturated electrolyte (i.e. 6.8 pH). A pair of bottom and top chips was assembled at the tip of a TEM holder (Hummingbird Scientific). The first dry cell was inserted into the TEM to confirm the position of the sample on the membrane/electrode and adjust the TEM alignment. Thereafter, the liquid electrolyte was infused into the cell by hand using a syringe, after which the syringe was released and disconnected from the holder. This manipulation favours the formation of a wetting layer inside the liquid cell. Experiments were performed in a static cell configuration to avoid filling all the space between the membranes when flowing the electrolyte. The experimental footprint of bubbles on the chronoamperometry profile shows that the bubbles dissipate/collapse after formation. However, despite being dissipated, this process may cause movement of particles, which could result in a change to the measured projection area. Our observations of the restructuring processes take place before bubble formation, which eliminates the effect of the bubbles on these processes.

All electron imaging was done on a JEOL 2200FS TEM equipped with an omega energy filter. Energy-filtered TEM mode was obtained by inserting a 10 eV energy-selecting slit and centering

it on the zero-loss peak of the electron energy loss spectrum. Image acquisition was done with a direct electron camera (DE16, Direct Electron) with a frame rate of 20 frames per second.

Electrochemical measurements were performed using SP-300 (BioLogic France) equipped with ultra-low current cable. LSV was done at 50 mV/s scan rate from open circuit potential to -0.8 V vs RHE. Chronoamperometry was measured at -0.8 V vs RHE for 5 min and was set to start automatically after the LSV measurement.

Data post-processing

Image denoising

For denoising using the cryoCARE workflow, training pairs were generated by splitting the raw image sequence into subsets of even and odd numbers to obtain statistically independent noise-limited volumes. A total of 2000 training volumes and 200 validation volumes were used. Each volume had dimensions of 64 x 64 x 64 pixels. The network was trained for 100 epochs with 62 steps per epoch using batch size of 32.

Image segmentation

Prior to segmentation, a new subsequence of images was generated by extracting images at 50-slice intervals from the denoised image sequence. The Segment Anything Model 2 (SAM2) plugin in ImageJ was then used to detect and outline particles in the denoised image sequence. To improve segmentation accuracy, SAM2 Large variant, the largest available pretrained model, was selected. Segmentation was performed using a prompt-based approach, in which bounding box prompts were manually defined around each particle of interest. After segmentation, the enclosed area of each particle was measured for every extracted slice. These measurements were then exported and saved as text files for subsequent analysis.

Particle analysis

The histogram in the inset of Figure 1a was generated by segmenting five HAADF STEM images containing a total of 129 representative Cu nanocubes (Figure S1). The mean projected area and standard deviation were calculated using column statistics analysis in Origin Lab. Plots of catalyst projection area were constructed by taking an average of 12 to 20 nanocubes' projection area and

error bars were calculated as the standard deviation of the respective sample number. Particle projection area distribution histograms were plotted using the projection area of all particles in the field of view for each time-point analyzed. The average dissolution rate for the nanocube in terms of projection area was calculated by dividing the change (decrease) in projection area from the beginning until the elapsed time.

To calculate the local curvature, the outline of all particles measured by segmentation in ImageJ were exported as a list of X and Y coordinates. The local curvature κ was calculated using the formula for the curvature of a parametric curve in 2D:

$$\kappa = \frac{|x'y'' - y'x''|}{(x'^2 + y'^2)^{1.5}},$$

where x', y' and x'', y'' are first and second order derivatives of the x and y components, respectively, all of which were calculated using the numpy.gradient function in Python. Higher κ values respectively correspond to larger curvature.

Chronoamperometry for the CO_2 electroreduction reaction (CO_2ER)

Electrochemical measurements were carried out in a custom gas-tight polycarbonate H-cell, sealed with Buna-N O-rings. The working and counter electrodes were positioned parallel to each other to maintain uniform potential across the working electrode surface. The anodic and cathodic compartments, separated by a Selemion AMV anion exchange membrane, each held 2 mL of electrolyte to concentrate liquid products. Chronoamperometry was performed at the desired potential with a steady CO_2 flow of 5 sccm. PEIS, LSV, and ECDL analyses were conducted before and after each chronoamperometry experiment. An 85% dynamic iR-compensation, based on PEIS-measured cell resistance, was applied throughout.

Products quantification

Gas products were separated and analyzed using a gas chromatograph (GC, SRI Instruments) equipped with a HayeSep D porous polymer column and both thermal conductivity and flame ionization detectors. The exhaust from the catholyte compartment was connected directly to the GC inlet. Calibration for H_2, CO, CH_4, C_2H_4, and C_2H_6 was carried out using five standard gas mixtures (Carbagas) to generate calibration curves for product concentration determination. Ultra-high purity N_2 (99.999%) was used as the carrier gas.

After electrolysis, liquid products were collected from both the catholyte and anolyte compartments to account for possible crossover—such as formic and acetic acids, which exist as anions (formate and acetate) and can migrate through the anion exchange membrane separating the compartments. The products were separated and quantified by high-performance liquid chromatography (HPLC) with a refractive index detector, using an Aminex HPX-87H (BioRad) column and a 5 mM H_2SO_4 eluent. Calibration curves for ethanol, n-propanol, ethylene glycol, formic acid, and acetic acid were prepared in a 0.1 M $KHCO_3$ electrolyte.

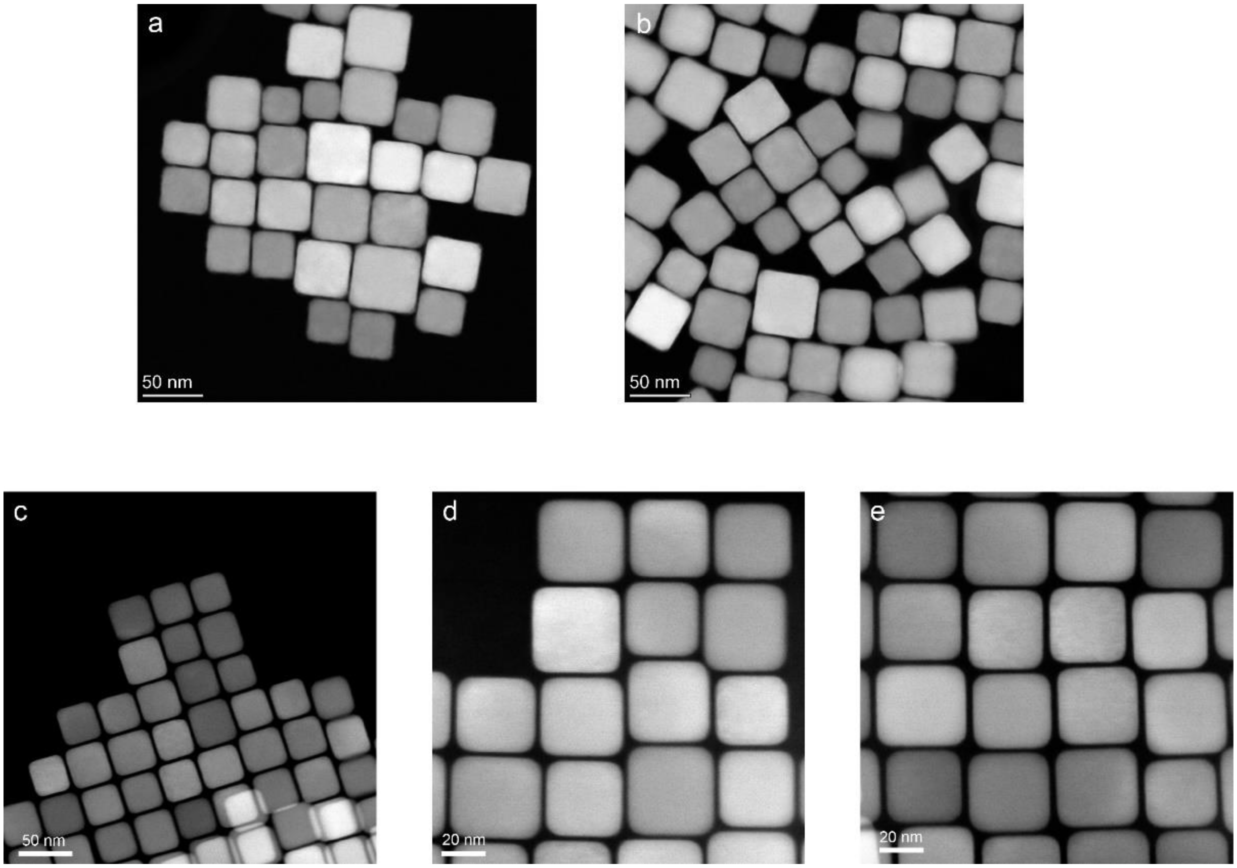

Supplementary Figure S1. (a)-(e) HAADF STEM images of pristine Cu NCs used to generate the histogram in Figure 1a.

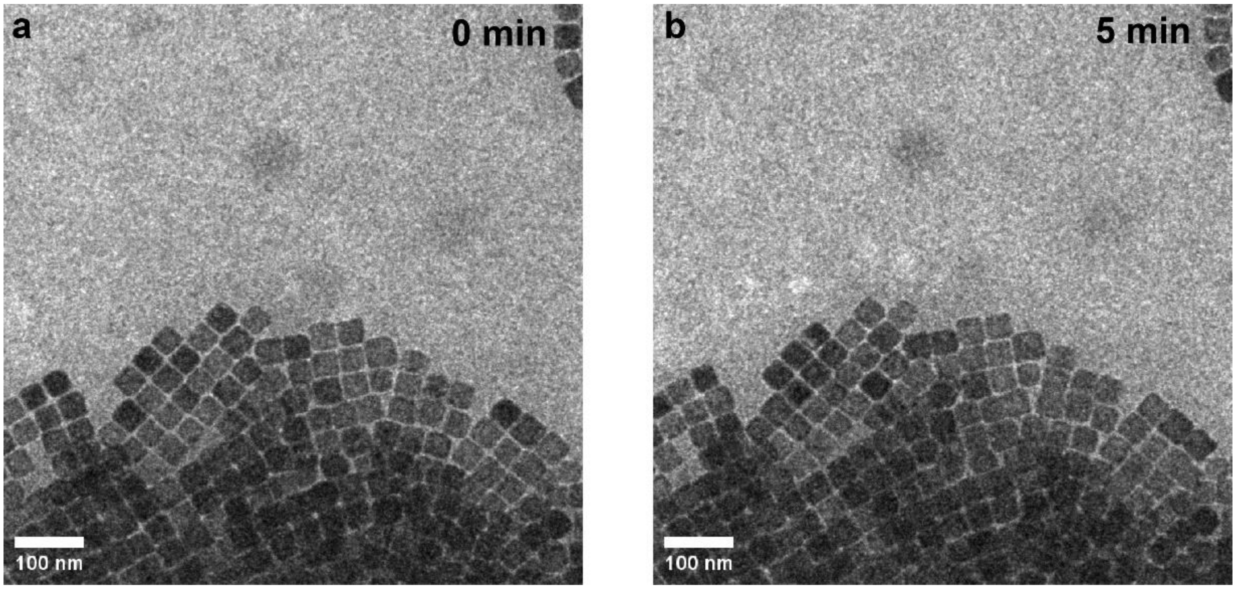

Supplementary Figure S2. LPTEM control experiments without applied potential. Cu NCs before (a) and after (b) continuous imaging at 60 $e^{-}nm^{-2}s^{-1}$.

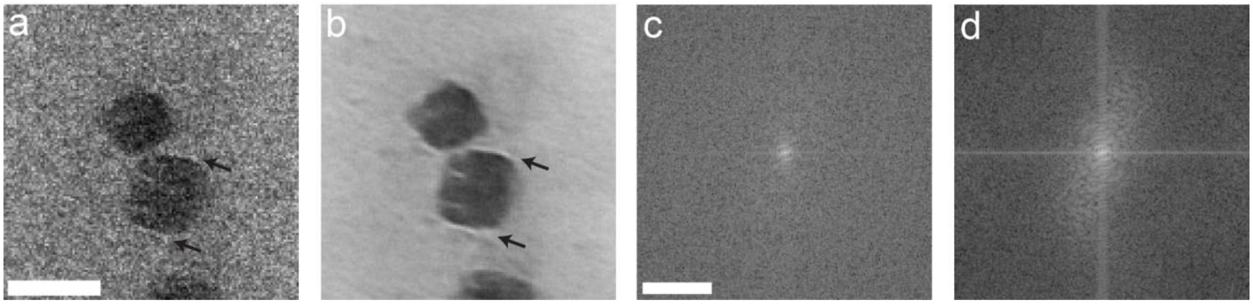

Supplementary Figure S3. (a) Raw image of Cu nanocubes obtained by averaging 20 subsequent frames to improve image quality. Scale bar, 50 nm. (b) CryoCARE denoised image of Cu nanocubes. Black arrows indicate on white contrast resulting from objective astigmatism, enhanced by the averaging. (c) and (d) Corresponding FFT patterns of images in (a) and (b), respectively. Scale bar, 0.2 nm-1. Raw TEM images were acquired at 10kX magnification with a pixel size of 1.16 nm/pix, corresponding to a field of view of 2048x2048 pix. Images in (a) and (b) correspond to 137x137 pix cropped regions. While for statistical analysis regions of interest containing individual or particle assemblies were cropped to sizes ranging from 95x95 to 158x158 pix, depending on the particles size and required background.

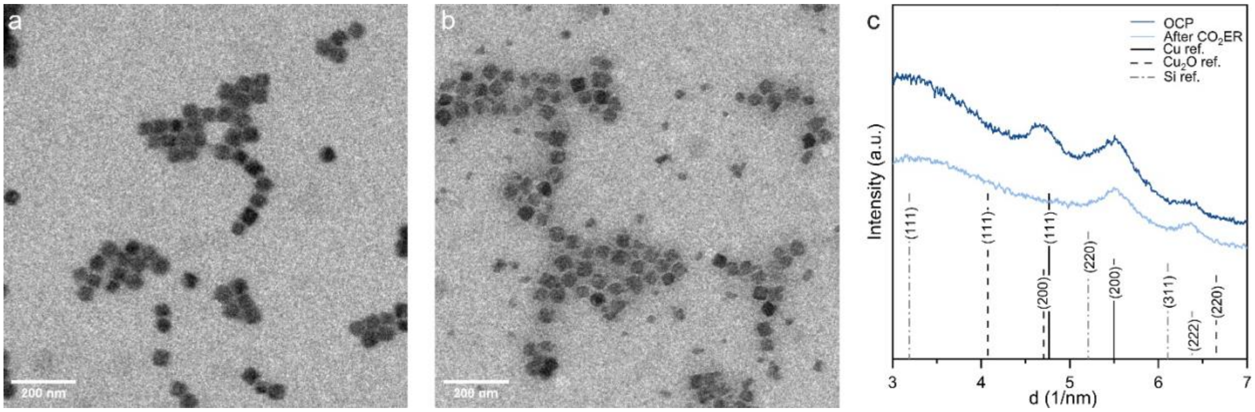

Supplementary Figure S4. (a) in situ EFTEM image of Cu NCs before and (b) after CO_2ER experiment. (c) Radial profile of SAED pattern taken before and after in situ TEM CO_2ER experiment.

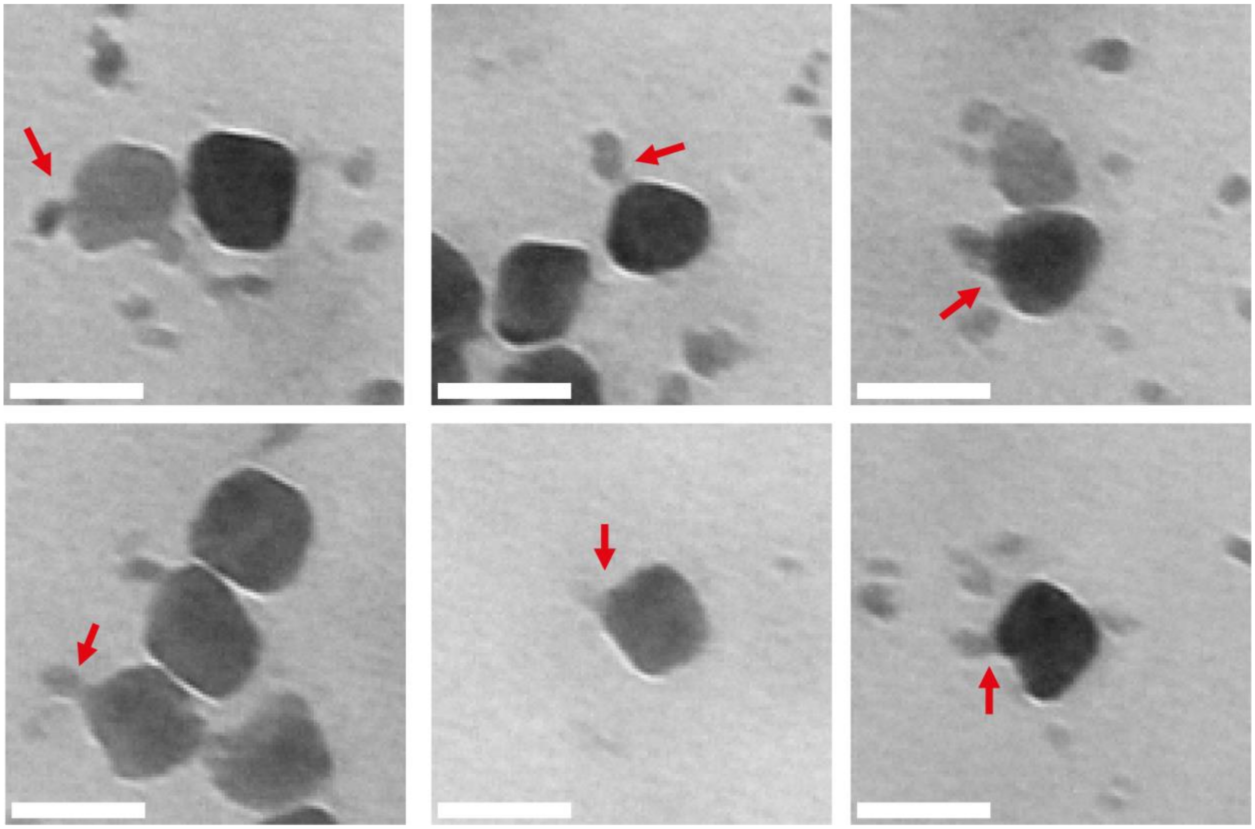

Supplementary Figure S5. In situ EFTEM denoised images of redeposited particles attached to the high-energy sites of Cu nanocubes. Scale bar, 50 nm.

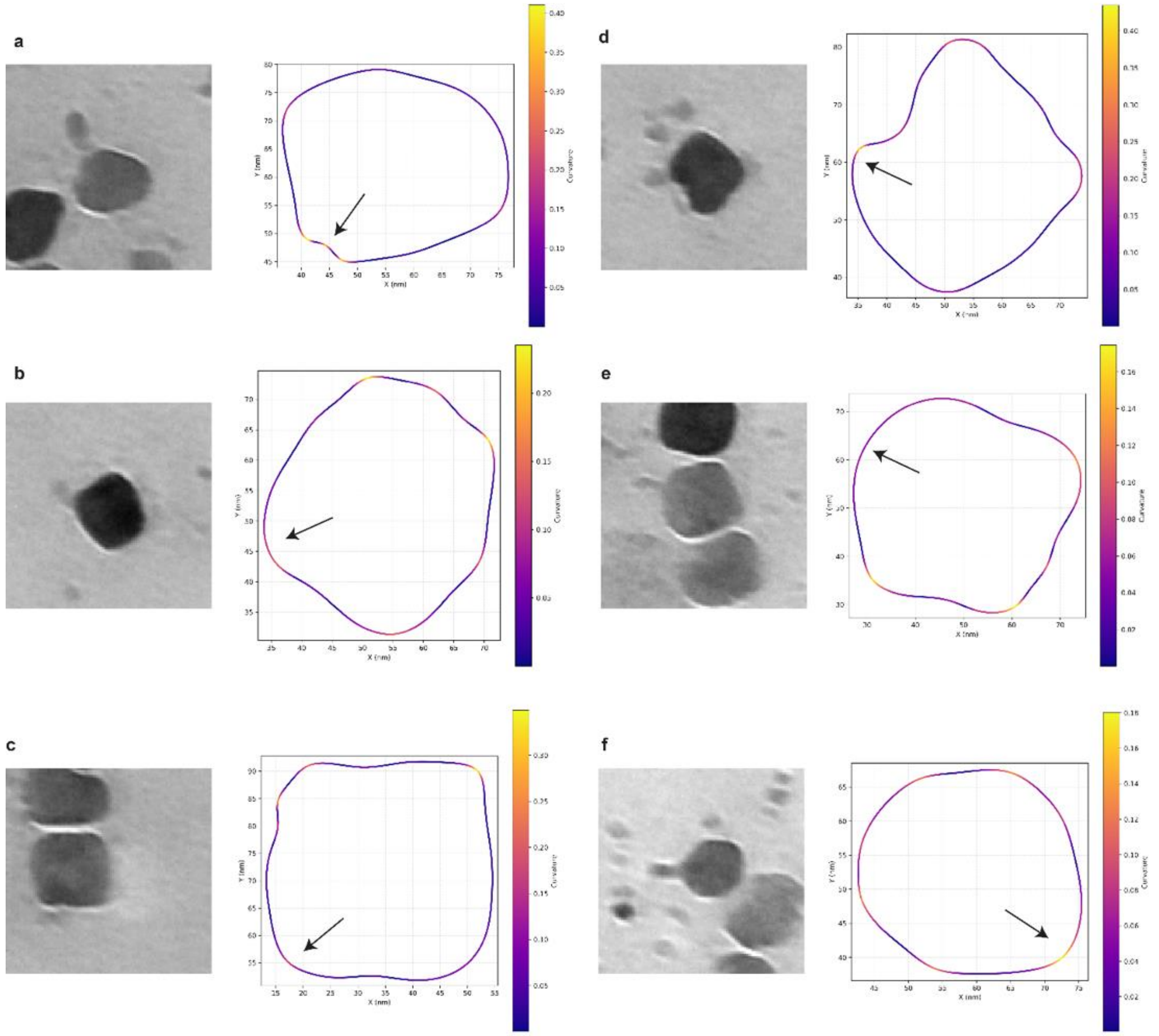

Supplementary Figure S6. (a)-(f) Outline and local curvature of nanocubes that underwent reattachment of redeposited nanoparticles. Black arrows indicates the attachment spots of the nanoparticles. All panels show denoised images.

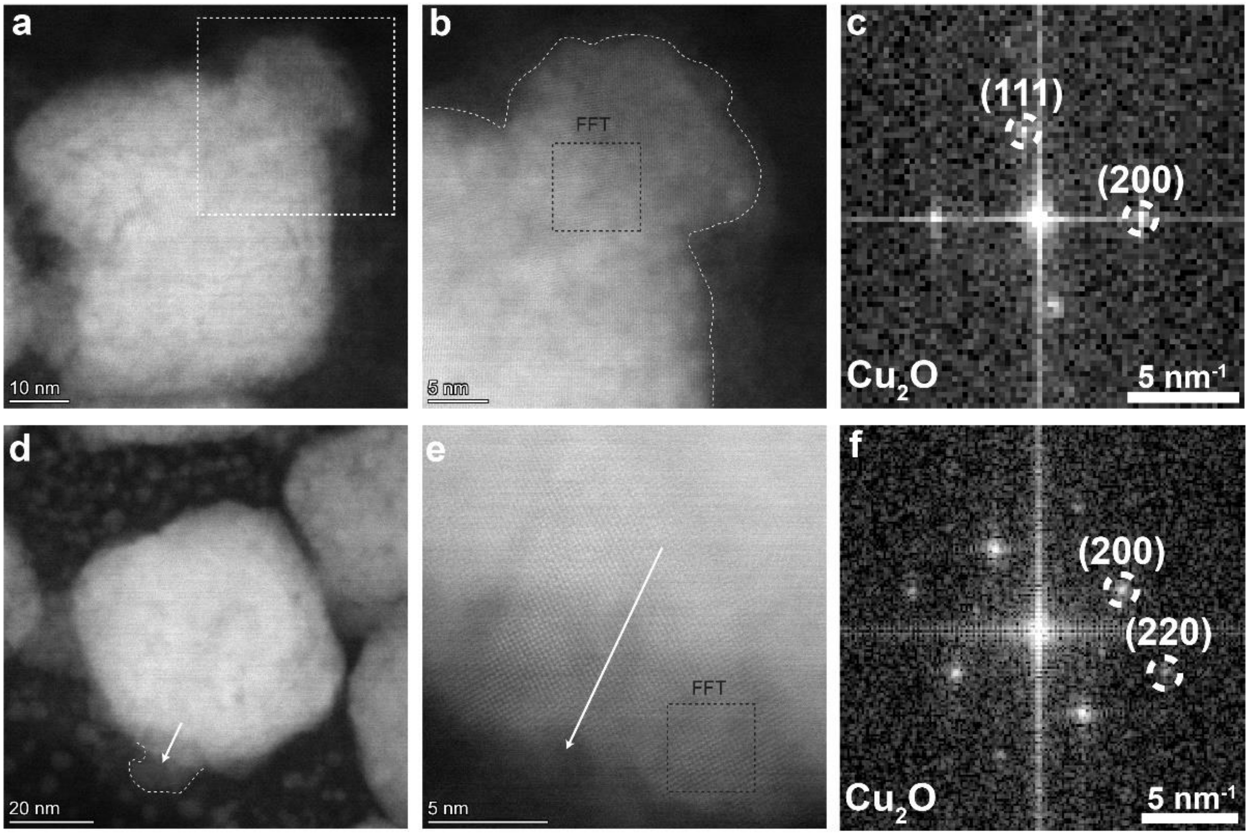

Supplementary Figure S7. Post-mortem high resolution (HR) HAADF STEM imaging of Cu nanocubes after LPTEM experiment. (a), (b) HR HAADF images of Cu NCs and (d), (e) zoomed-in images of selected corners. (c) and (f) Fast Fourier transforms (FFT) from square regions indicated in (b) and (e), respectively, indicate Cu_2O phase.

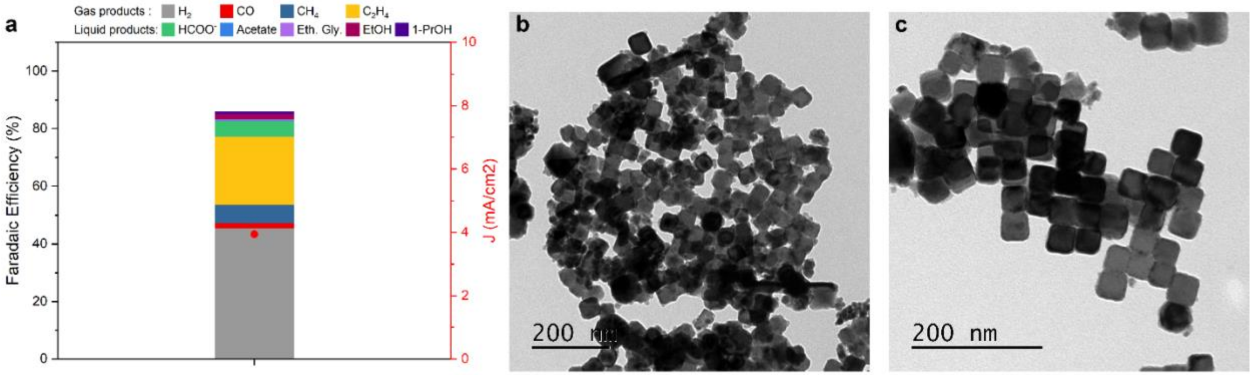

Supplementary Figure S8. (a) Faradaic efficiency and current density after two hours of CO_2ER at -1.1 V vs RHE in an H-cell configuration with a CO_2 saturated 0.1M $KHCO_3$ electrolyte. (b) and (c) Post-mortem TEM images taken after the two-hour CO_2ER measurement.

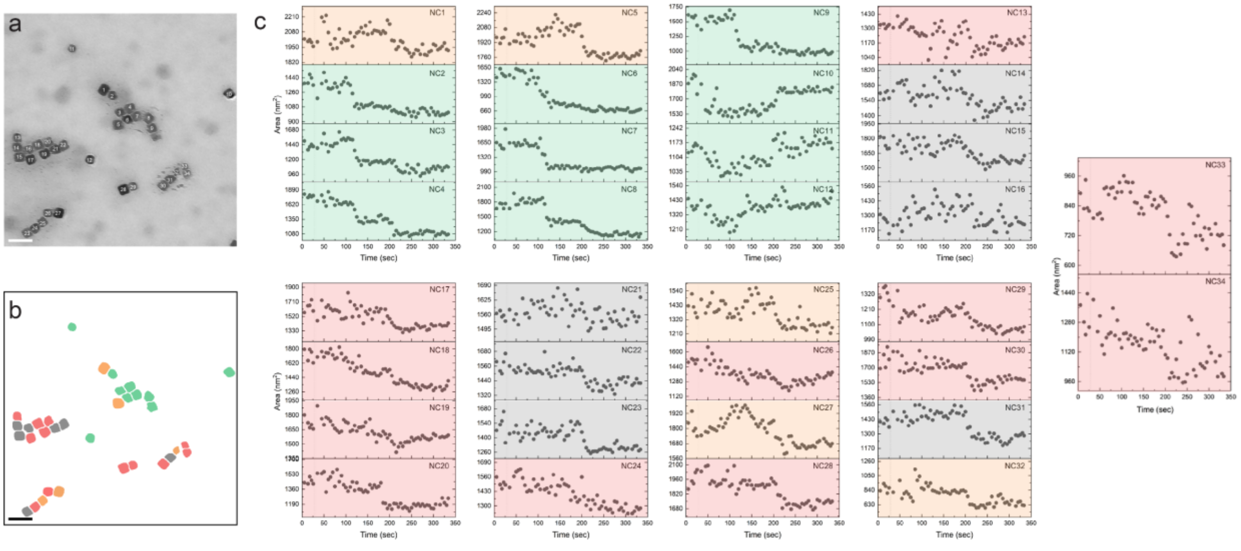

Supplementary Figure S9. (a) In situ EFTEM denoised image of Cu NCs with annotated particles. (b) Segmented and colored image. Scale bar, 100 nm. (c) The projection area of Cu NCs in (a) versus the time of the CO_2ER experiment. Profiles with similar behavior were colored in the same color. Red corresponds to dissolution only, green – to dissolution/fragmentation, orange – to dissolution/reattachment, and grey is assigned to particles with relatively stable projection area over time. The drop or jump in the projection area profile due to cell instability at around 200 s was not considered when assigning the behavior of particle restructuring.

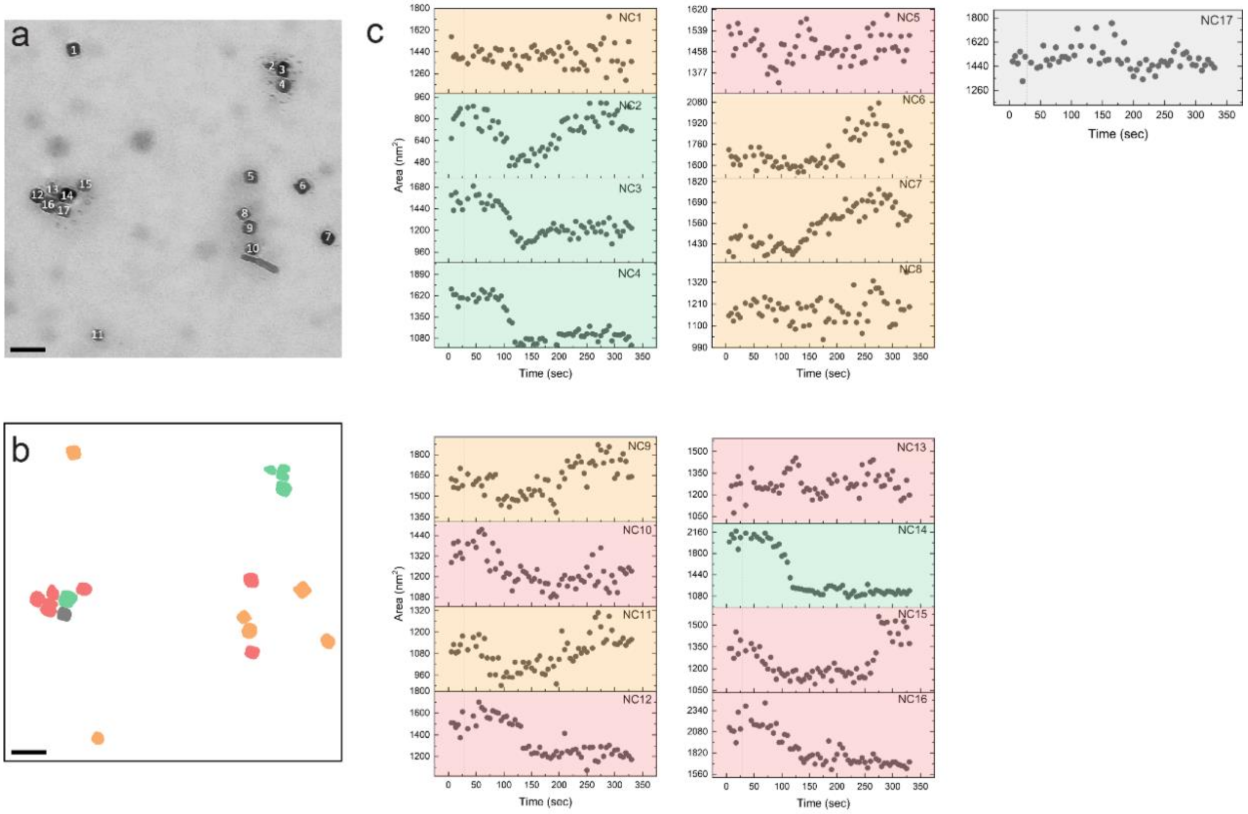

Supplementary Figure S10. (a) In situ EFTEM denoised image of Cu NCs with annotated particles in area 2. (b) Segmented and colored image. Scale bar, 100 nm. (c) The projection area of Cu NCs in (a) versus the time of the CO_2ER experiment. Profiles with similar behavior were colored in the same color. Red corresponds to dissolution only, green – to dissolution/fragmentation, orange – to dissolution/reattachment/growth, and grey is assigned to particles with relatively stable projection area over time.

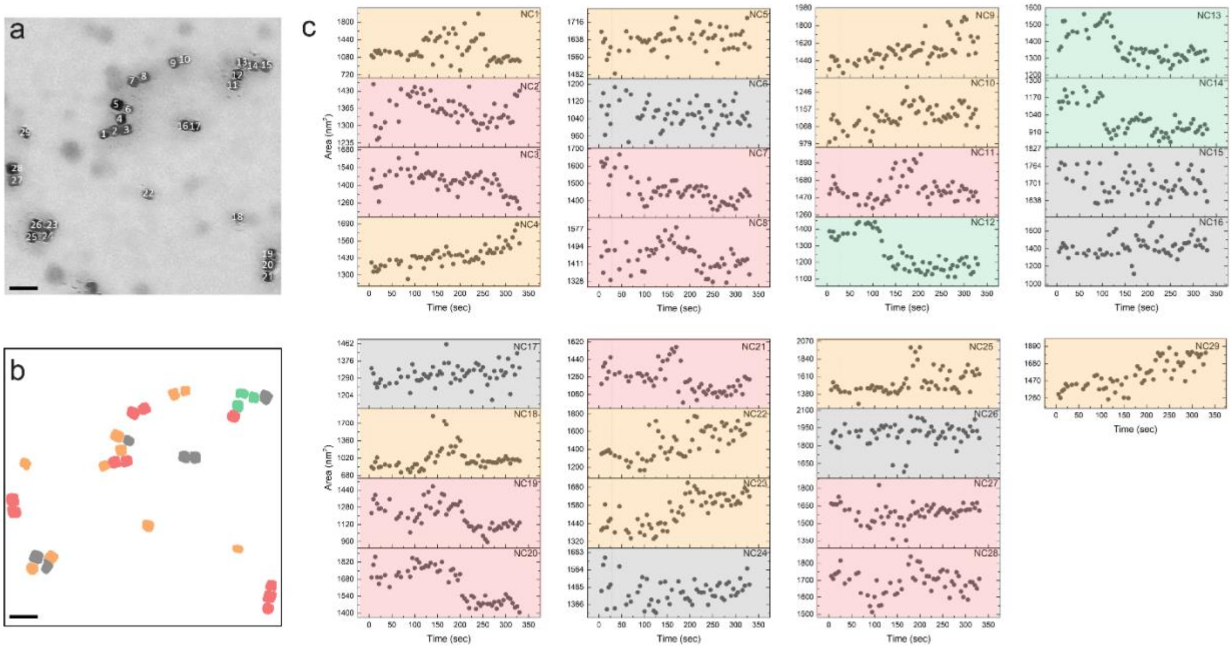

Supplementary Figure S11. (a) In situ EFTEM denoised image of Cu NCs with annotated particles in area 3. (b) Segmented and colored image. Scale bar, 100 nm. (c) The projection area of Cu NCs in (a) versus the time of the CO_2ER experiment. Profiles with similar behavior were colored in the same color. Red corresponds to dissolution only, green – to dissolution/fragmentation, orange – to dissolution/reattachment/growth, and grey is assigned to particles with relatively stable projection area over time.

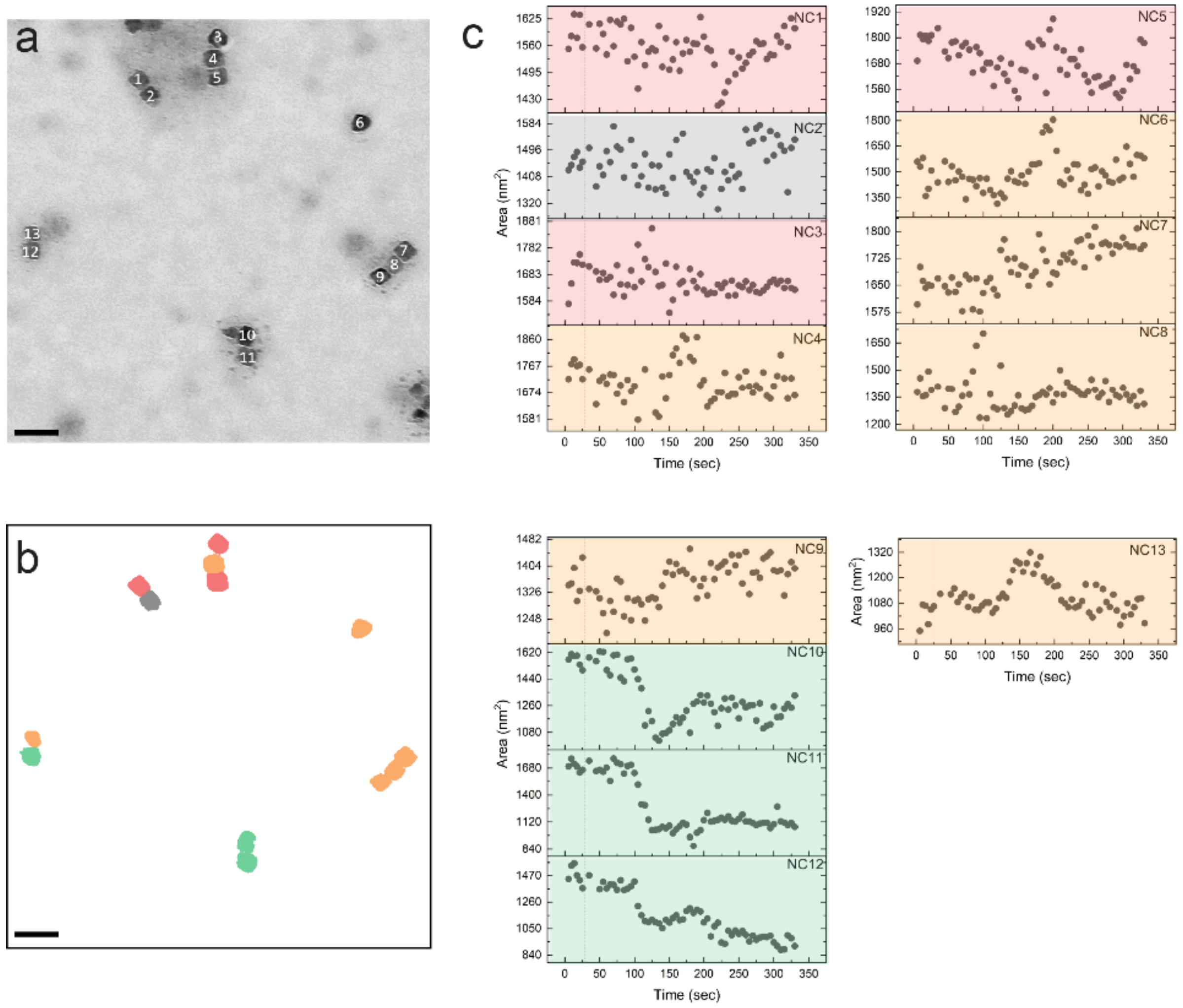

Supplementary Figure S12. (a) In situ EFTEM denoised image of Cu NCs with annotated particles in area 4. (b) Segmented and colored image. Scale bar, 100 nm. (c) The projection area of Cu NCs in (a) versus the time of the CO_2ER experiment. Profiles with similar behavior were colored in the same color. Red corresponds to dissolution only, green – to dissolution/fragmentation, orange – to dissolution/reattachment/growth, and grey is assigned to particles with relatively stable projection area over time.

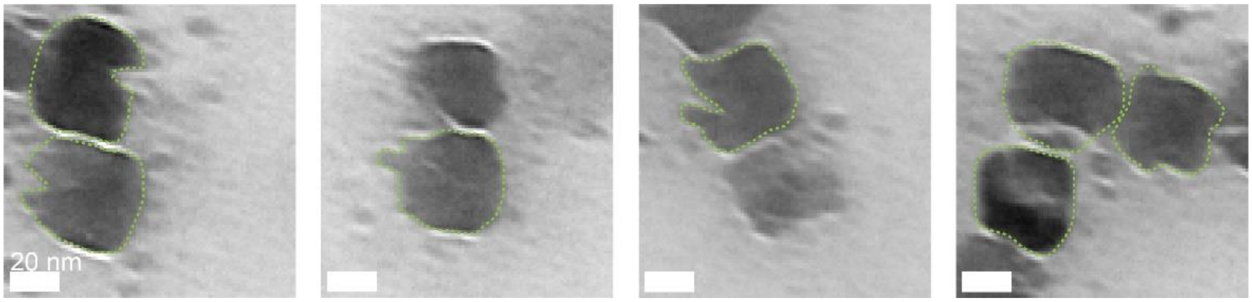

Supplementary Figure S13. Representative in situ TEM denoised images of Cu nanocubes before fragmentation, showing the distorted, irregular shape of the cubes. The green dashed line outlines the cubes that underwent fragmentation.

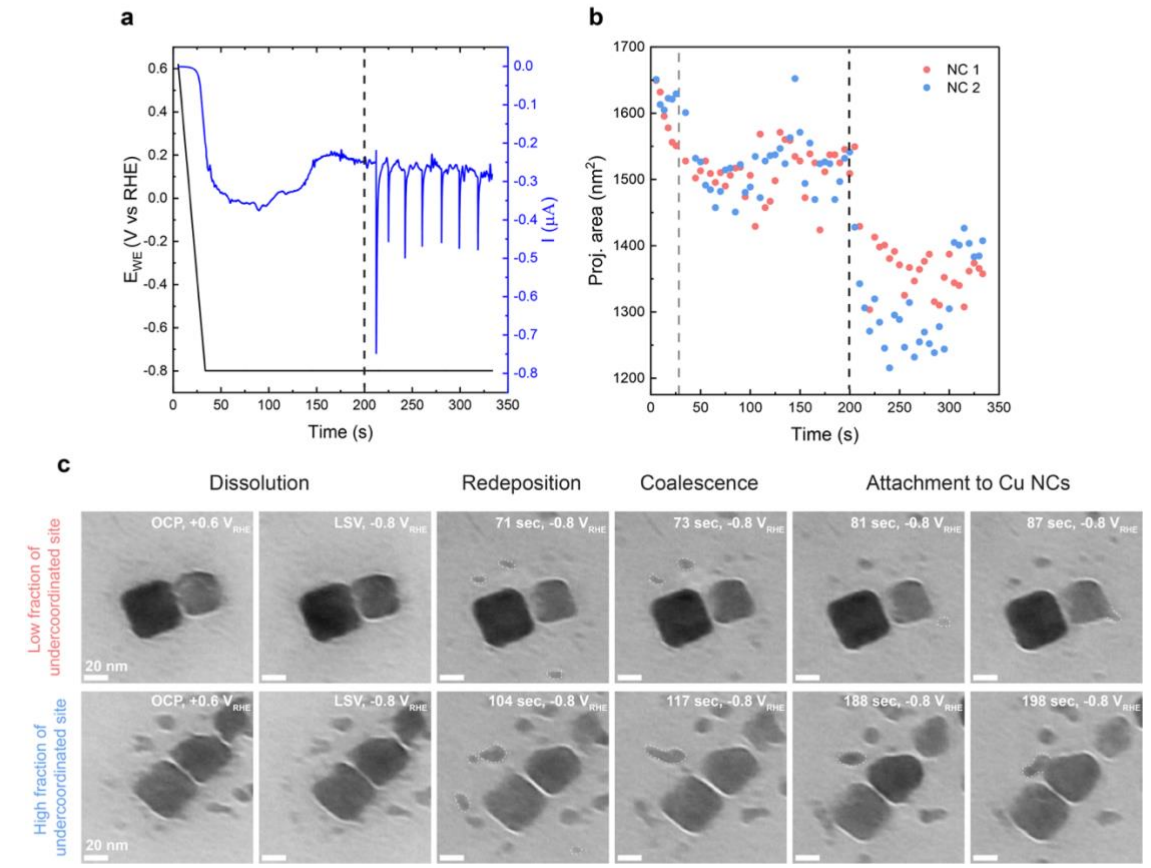

Supplementary Figure S14. Comparison of the degradation behavior of well-faceted Cu NCs and Cu NCs with a high fraction of undercoordinated sites. Panel (c) shows denoised images.

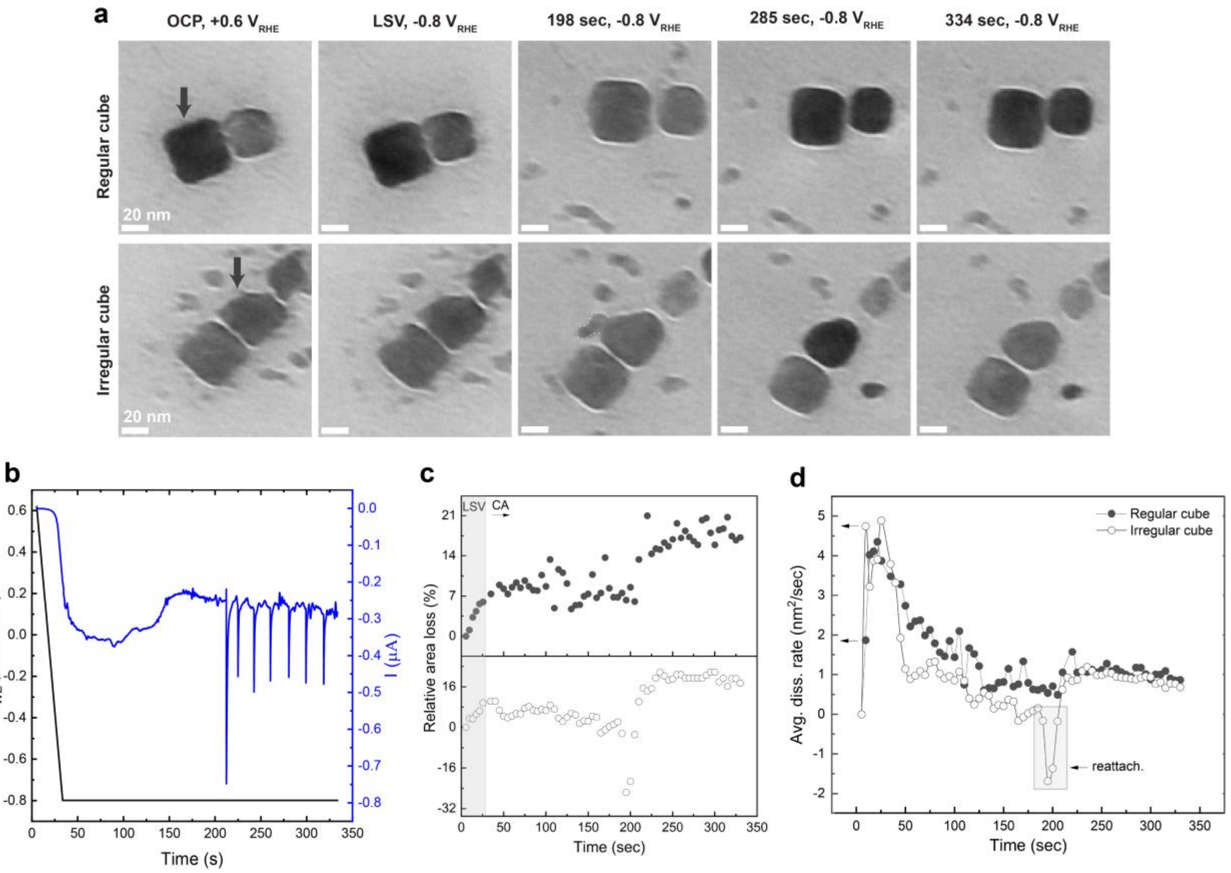

Supplementary Figure S15. Comparison of dissolution kinetics of a regular well faceted cube and an irregular distorted nanocube. (a) Time-lapse denoised image series of two representative cubes indicated by the black arrow. (b) Current and potential profiles during linear sweep voltammetry and chronoamperometry. (c) Relative area loss profiles for each nanocube. (d) Average dissolution rate profile for both nanocubes. The black arrow within the first 25 seconds indicates the difference in dissolution rate between the regular and irregular cubes at the beginning of the reaction. Panel (a) shows denoised images.

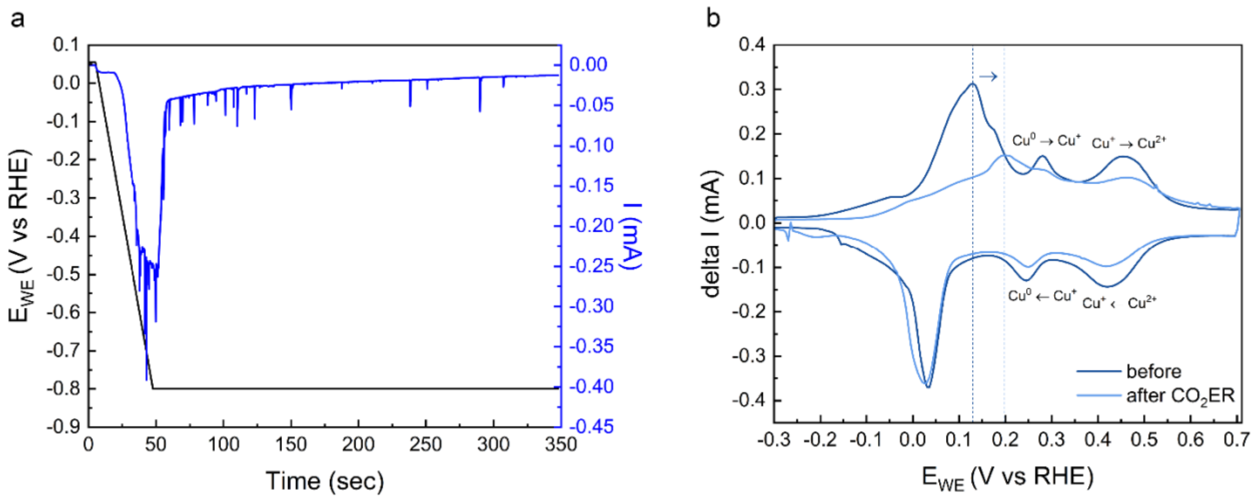

Supplementary Figure S16. (a) On bench LSV-CA measurement of Cu NCs at similar conditions as in situ TEM experiment. (b) Square wave voltammetry of Cu nanocubes right after the LSV-CA measurement.

Captions of Supporting Movies

Supplementary Movie S1 – Denoised TEM image recording of Cu NCs during LSV-CA at -0.8 V vs RHE_ playback at 100fps

Supplementary Movie S2 – Denoised TEM image recording of Cu NCs during LSV-CA at -0.8 V vs RHE_ playback at 100fps_scale_bar_20nm

Supplementary Movie S3 – Denoised TEM image recording of Cu NCs during LSV-CA at -0.8 V vs RHE_ playback at 100fps_area2

Supplementary Movie S4 – Denoised TEM image recording of Cu NCs during LSV-CA at -0.8 V vs RHE_ playback at 100fps_area3

Supplementary Movie S5 – Denoised TEM image recording of Cu NCs during LSV-CA at -0.8 V vs RHE_ playback at 100fps_area4
